# Supplementary figures and images for: Cryo-EM Structure of the Type IV Pilus Extension ATPase from Enteropathogenic Escherichia coli
Source: mBio. 2022 Nov 3;13(6):e02270-22. doi: 10.1128/mbio.02270-22 (PMC9765406; doi:10.1128/mbio.02270-22)

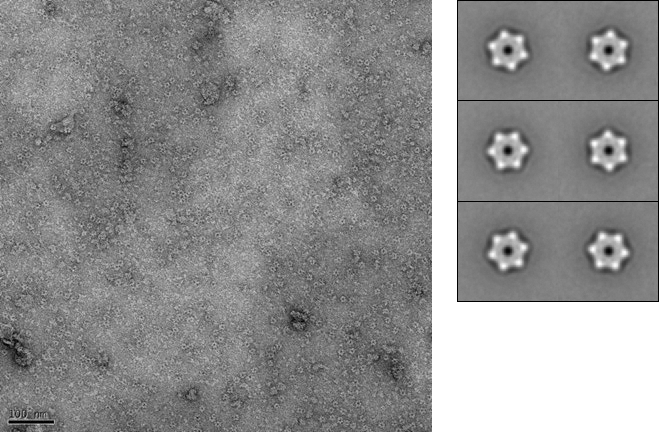

Supplement: FIG S1 [file mbio.02270-22-s0001.tif]

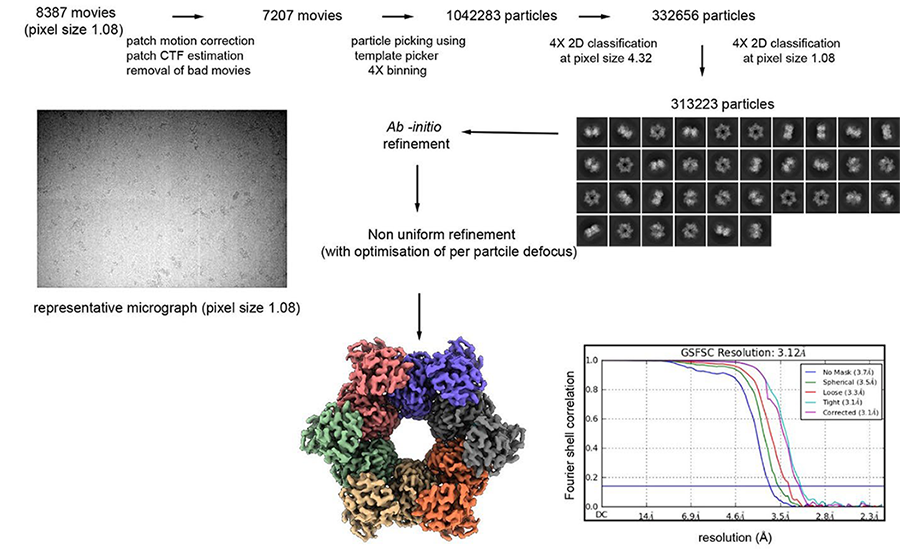

Supplement: FIG S2 [file mbio.02270-22-s0002.tif]

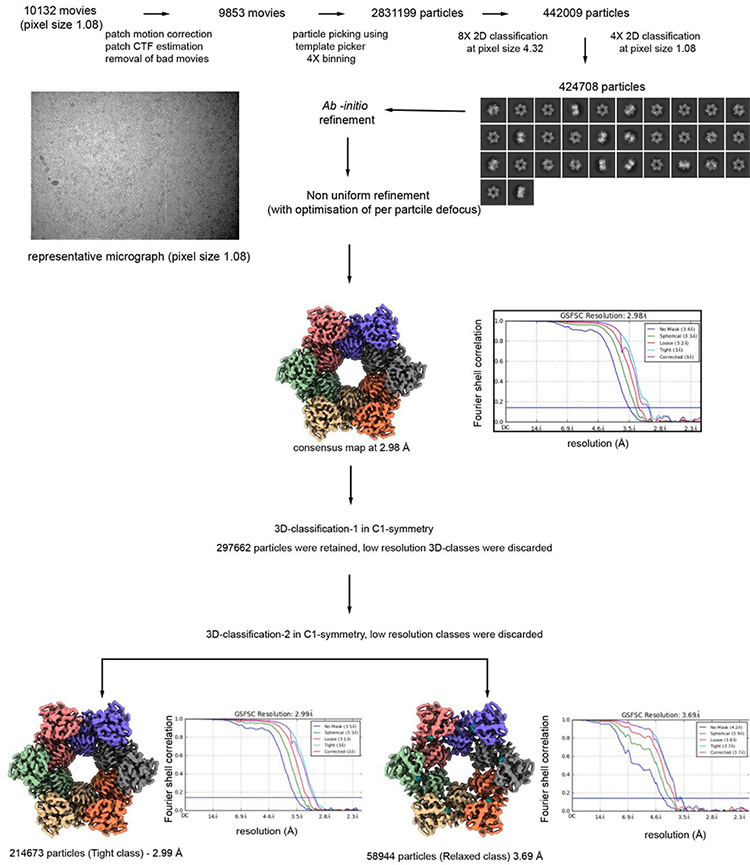

Supplement: FIG S3 [file mbio.02270-22-s0003.tif]

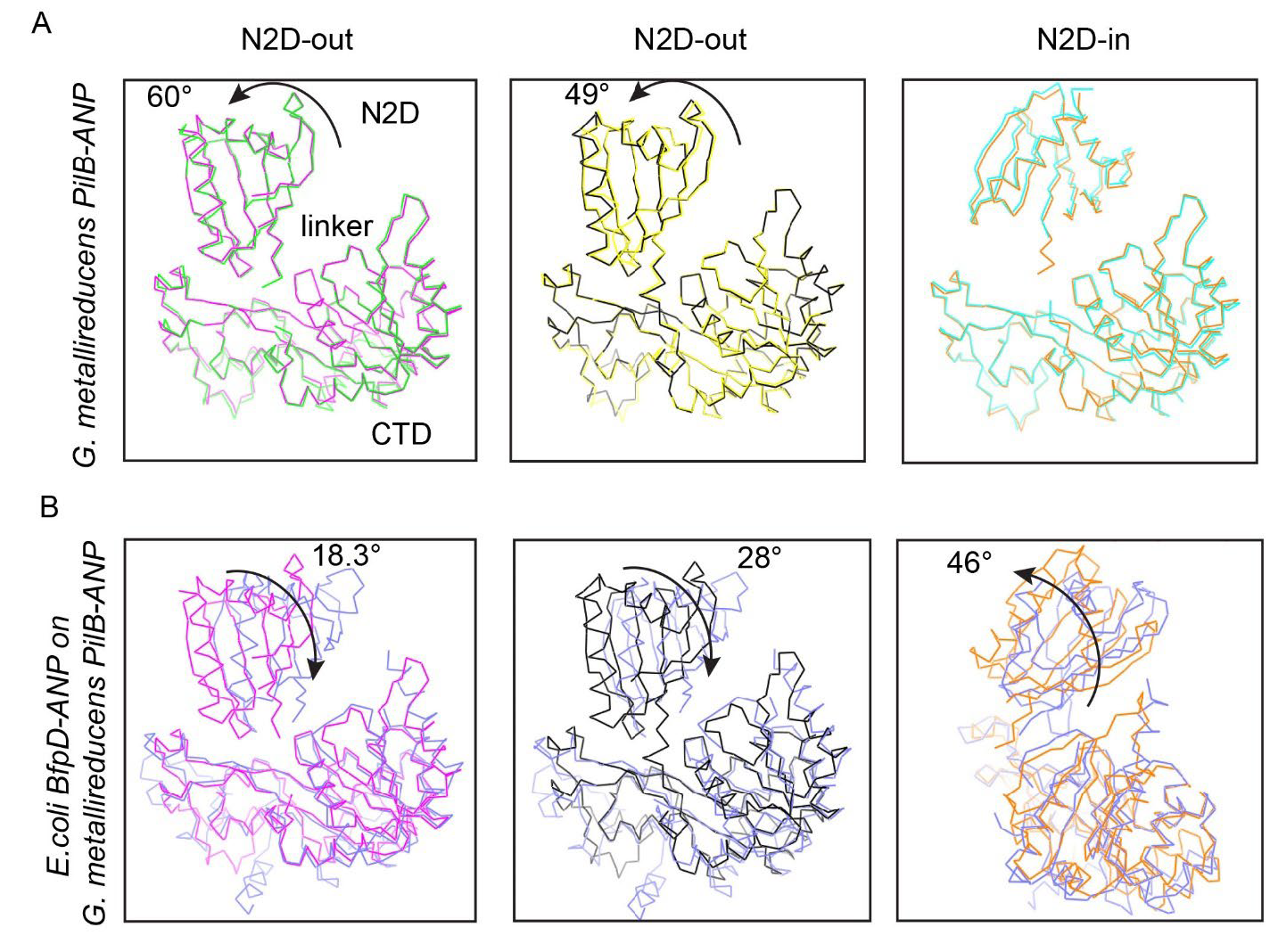

Supplement: FIG S4 [file mbio.02270-22-s0004.tif]

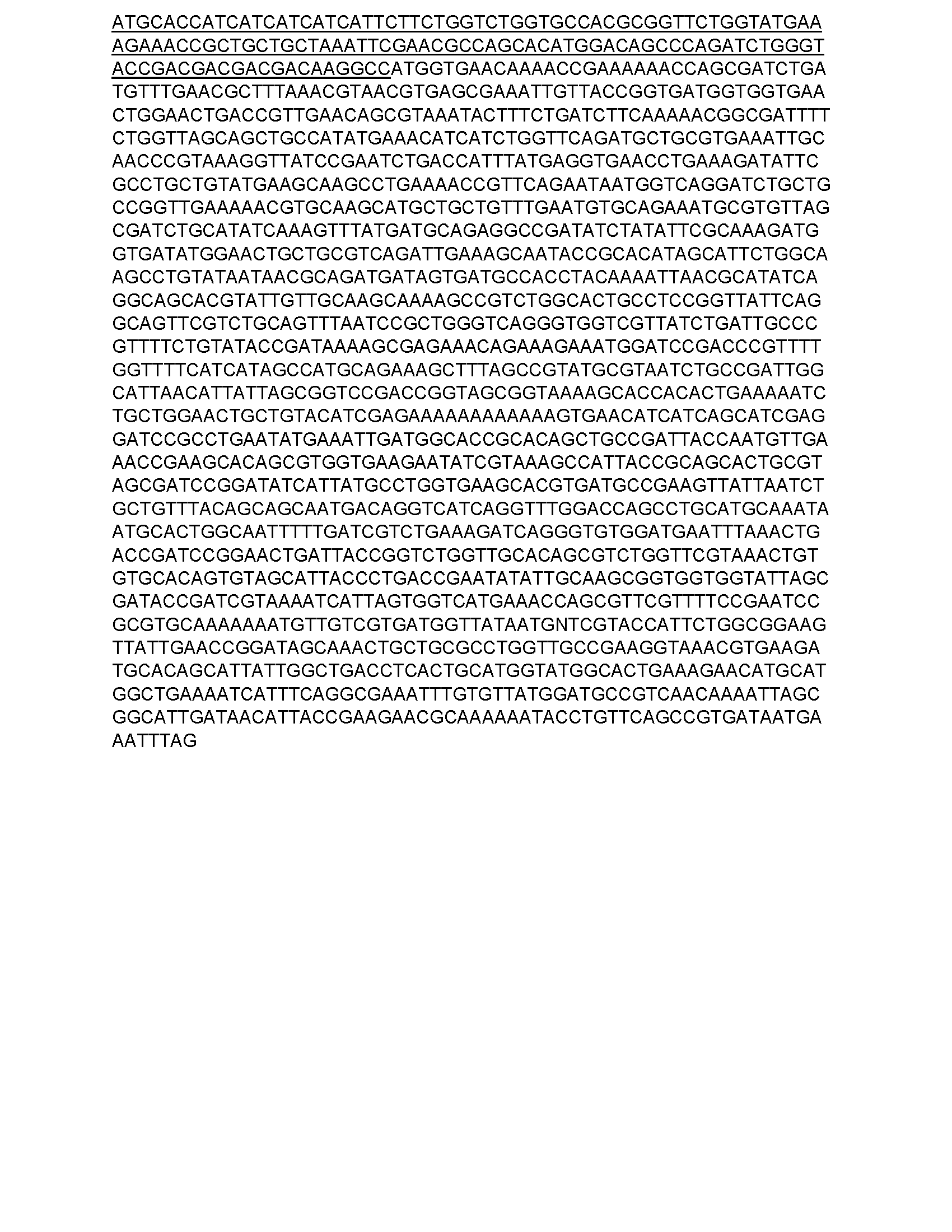

Supplement: FIG S5 [file mbio.02270-22-s0005.tif]
