# Supplementary material for: Cryo-EM Structure of the Type IV Pilus Extension ATPase from Enteropathogenic Escherichia coli
Source: mBio. 2022 Nov 3;13(6):e02270-22. doi: 10.1128/mbio.02270-22 (PMC9765406; doi:10.1128/mbio.02270-22)
Supplement: TABLE S2 [file mbio.02270-22-s0009.docx]

**Table S2.** Strains and plasmids used in this study

| **Strain or Plasmid** | **Genotype or description** | **Source or reference** |
| --- | --- | --- |
| **Strains** |  |  |
| DH5α | *supE44 ΔlacU169(φ80 lacZΔM15) hsdR17 recA1 endA1 gyrA96 thi-1 relA1* | Invitrogen |
| BL21(DE3) | F^–^ *omp*T *hsd*S_B_ (r_B_^–^, m_B_^–^) *gal dcm*(DE3) | Invitrogen |
| E2348/69 | Nalidixic acid-resistant variant of prototypic virulent O127:H6 clinical EPEC isolate | (1) |
| UMD926 | E2348/69 *bfpD::aphA3* | (2) |
|  |  |  |
| **Plasmids** |  |  |
| pET30a | Expression plasmid; Km^r^ | Novagen |
| BfpD-Hcp1 | Contains codon optimized *bfpD* gene fused to Hcp1 | Kurt Piepenbrink |
| pJZM005 | Codon-optimized *bfpD* in pET30a | This study |
| pJZM032 | pJZM031 with BfpD_E295C_ | This study |
| pJZM042 | pJZM031 with BfpD_E295C, E338Q_ | This study |
| pWKS30 | Low copy number vector; Ap^r^ | (3) |
| pRPA405 | Plasmid having *bfpD* gene with N-terminus His tag and S tag | (4) |
| pEMM1 | *bfpD* subcloned into pWKS30 but lacking its native stop codon. | This study |
| pJZM031 | Wild type *bfpD* complementation plasmid with corrected stop codon from pEMM1 | This study |
| pJZM032 | pJZM031 with BfpD_E295C_ | This study |
| pJZM036 | pJZM031 with BfpD_E338Q_ | This study |

1. Nisa, S., Hazen, T. H., Assatourian, L., Nougayrede, J. P., Rasko, D. A., and Donnenberg, M. S. (2013) In vitro evolution of an archetypal enteropathogenic *Escherichia coli* strain. *J Bacteriol* **195**, 4476-4483

2. Anantha, R. P., Stone, K. D., and Donnenberg, M. S. (2000) Effects of *bfp* mutations on biogenesis of functional enteropathogenic *Escherichia coli* type IV pili. *J Bacteriol* **182**, 2498-2506

3. Wang, R. F., and Kushner, S. R. (1991) Construction of versatile low-copy-number vectors for cloning, sequencing and gene expression in *Escherichia coli*. *Gene* **100**, 195-199

4. Crowther, L. J., Anantha, R. P., and Donnenberg, M. S. (2004) The inner membrane subassembly of the enteropathogenic Escherichia coli bundle-forming pilus machine. *Mol Microbiol* **52**, 67-79
