# Supplementary material for: Cryo-EM Structure of the Type IV Pilus Extension ATPase from Enteropathogenic Escherichia coli
Source: mBio. 2022 Nov 3;13(6):e02270-22. doi: 10.1128/mbio.02270-22 (PMC9765406; doi:10.1128/mbio.02270-22)
Supplement: TABLE S3 [file mbio.02270-22-s0010.docx]

**Table S3.** Primers used in this study.

| **Name** | **Sequence* (5` - 3`)** | **Target** | **Purpose** |
| --- | --- | --- | --- |
| BfpDNcoI | TTGATCCCTTCA**CCATGG**TG | *bfpD* | Amplification of *codon optimized bfpD gene* |
| BfpDXhoI | TGACGT**CTCGAG**ctaaatttcattatcacggctgaac | *bfpD* |  |
| BfpD E295C.P1 | gatccgcct**tgc**tatgaaattgatggcaccgcac | *bfpD* | *For creation of bfpD*_E295C_ |
| BfpD E295C.P2 | aatttcata**gca**aggcggatcctcgatgctg | *bfpD* |  |
| E338Q.P1 | cacgtgc**ctg**accaggcataatgatatccgg | *bfpD* | *For creation of bfpD*_E338Q_ |
| E338Q.P2 | tgcctggt**cag**gcacgtgatgccgaagttat | *bfpD* |  |
| pEMM1TAG.P1 | aacgagataTAGGAATTCGAGCTCCAATTCG | *bfpD* | Stop codon TAG insertion in pEMM1 Making plasmid pJZM031 |
| pEMM1TAG.P2 | GAGCTCGAATTCCTAtatctcgttatctctgctg | *bfpD* |  |
| wtbfpDE295C.P1 | tcaatttcatagcaaggtggatcttcaatgctg | *bfpD* | *bfpD*_E295C_ for complementation. Making plasmid pJZM032 |
| wtbfpDE295C.P2 | atccaccttgctatgaaattgacggcacggc | *bfpD* |  |
| wtbfpDE338Q.P11 | AGCTTGATATCGAATTCCTGC | *bfpD* | *bfpD*_E338Q_ for complementation. Making plasmid pJZM036 |
| wtbfpDE338Q.P2 | ccctggcctgccctggcattattatatccgg | *bfpD* |  |
| wtbfpDE338Q.P1 | gccagggcaggccagggatgctgaagtga | *bfpD* |  |
| wtbfpDE338Q.P22 | ACTATAGGGCGAATTGGAGC | *bfpD* |  |

*****Nucleotides underlined and with bold font indicate restriction sites or mutated codons. Gene coding nucleotides are indicated by lowercase letters, while uppercase letters highlight plasmid based or additional exogenous nucleotides added to facilitate cloning of the specified gene.
