# Supplementary material for: Cryo-EM Structure of the Type IV Pilus Extension ATPase from Enteropathogenic Escherichia coli
Source: mBio. 2022 Nov 3;13(6):e02270-22. doi: 10.1128/mbio.02270-22 (PMC9765406; doi:10.1128/mbio.02270-22)
Supplement: TABLE S1 [file mbio.02270-22-s0008.docx]

**Table S1.** Summary of cryo-EM image processing and model building statistics

|  | **BfpD-ADP** | **BfpD-ANP (class-1)** |  | **BfpD-ANP (class-2)** |
| --- | --- | --- | --- | --- |
| **Data Acquisition** |  |  |  |  |
| Microscope/Detector | Krios/K3 |  | Krios/K3 |  |
| Voltage (kV) | 300 |  | 300 |  |
| Magnification | 81000 |  | 81000 |  |
| Data collection mode | Counting |  | Counting |  |
| Pixel Size (Å) (super-resolution) | 1.08 |  | 1.08 |  |
| Focus range (μm) | -1. to -2.25 |  | -1. to -2 |  |
| Total electron dose (e/Å^2^) (Number of frames) | 60 (40) |  | 60 (40) |  |
| Total number of movies | 8387 |  | 10132 |  |
| **Image Processing** |  |  |  |  |
| Total number of particles picked | 1042283 |  | 2831199 |  |
| Particles after 2D classification | 332656 |  | 442009 |  |
| Particles used for 3D refinement | 313223 | 214673 |  | 68944 |
| Resolution (Å) | 3.12 | 2.99 |  | 3.69 |
| EMDB ID | 27797 | 27795 |  | 27796 |
| **Model Refinement** |  |  |  |  |
| RMS Deviation (Bonds) | 0.003 | 0.002 |  | 0.002 |
| RMS Deviation (Angle) | 0.563 | 0.625 |  | 0.598 |
| Ramachandran Plot statistics (%) |  |  |  |  |
| Preferred | 94.95 | 92.84 |  | 93.58 |
| Allowed | 4.81 | 6.91 |  | 6.17 |
| Outliers | 0.24 | 0.25 |  | 0.25 |
| **Model Validation** |  |  |  |  |
| Clash-score | 2.92 | 4.1 |  | 5.77 |
| MolProbity Score | 1.43 | 1.65 |  | 2.10 |
| PDB ID | 8DZG | 8DZE |  | 8DZF |
